# Effect of the presence of the articular cartilage on the femoral component rotation in total knee arthroplasty in female and varus osteoarthritis knees

**DOI:** 10.1186/s13018-020-02030-9

**Published:** 2020-10-29

**Authors:** Ji-Hoon Nam, Yong-Gon Koh, Paul Shinil Kim, Joon-Hee Park, Kyoung-Tak Kang

**Affiliations:** 1 Department of Mechanical Engineering, Yonsei University, 50 Yonsei-ro, Seodaemun-gu, Seoul, 03722 Republic of Korea; 2 Joint Reconstruction Center, Department of Orthopaedic Surgery, Yonsei Sarang Hospital, 10 Hyoryeong-ro, Seocho-gu, Seoul, 06698 Republic of Korea; 3 Department of Orthopaedic Surgery, The Bone Hospital, 67, Dongjak-daero, Dongjak-gu, Seoul, Republic of Korea; 4 Department of Anesthesiology & Pain Medicine, Hallym University College of Medicine and Kangdong Sacred Heart Hospital, 150 Seongan-ro, Gangdong-gu, Seoul, 05355 Republic of Korea

**Keywords:** Korean patients, Articular cartilage, Cartilage thickness, Femoral rotation, Varus knee

## Abstract

**Purpose:**

Surgical techniques for total knee arthroplasty (TKA) require femoral rotational corrections that alter the position of the surface of the posterior femoral joint especially in kinematic alignment. However, preoperative planning of TKA based on computed tomography (CT), without knowing the femoral cartilage thickness, may cause post-surgery failures in femoral rotation. Therefore, this study aimed to evaluate the effects of posterior condyle cartilage thickness on rotational alignment in the femoral component.

**Methods:**

Three-dimensional magnetic resonance imaging (MRI) scans were obtained for 139 male and 531 female osteoarthritis patients. The angles defined by the femoral posterior condylar axis (PCA) and the surgical transepicondylar axis (TEA) were evaluated with respect to the presence of cartilage. Additionally, these effects were evaluated with respect to patient gender and varus/valgus condition.

**Results:**

In all patients, the angle between the TEA and PCA was significantly greater in the presence of cartilage than in the absence of cartilage. This result was also seen in female patients. However, there was no difference in the TEA/PCA angle in male patients based on the presence of cartilage. The TEA/PCA angle was significantly greater in the presence of cartilage than in the absence of cartilage in the female varus group. However, there were no differences in the TEA/PCA angle based on the presence of cartilage in the male varus/valgus and female valgus groups. Cartilage thickness in the posterior femoral condyle was significantly greater on the lateral side than on the medial side in all and male patients. However, there was no difference between the genders regarding cartilage thickness.

**Conclusion:**

Surgical planning for TKA based on CT does not consider articular cartilage and could lead to external malrotation of the femoral implant. Therefore, the effect of the remaining posterior condylar cartilage should be considered by surgeons to prevent over-rotation of the femoral component, especially in female varus knees.

## Introduction

Proper alignment of the femoral component during rotation plays an important role in knee stability during flexion and patellofemoral kinematics [1]. Malalignment during rotation causes patellofemoral complications and poor ligament balancing, which may cause the failure of total knee arthroplasty (TKA) [2]. Many methods have been developed to assess femoral component rotation. The commonly used traditional methods for femoral rotational alignment in TKA involve the use of the Whiteside line, which is considered in reference to the surgical or clinical transepicondylar axis (TEA) and 3° external rotation of the posterior condylar axis (PCA) [3–5]. Theoretically, the TEA is a reliable reference axis for the proper location of the femoral component, but it is challenging to identify the TEA intraoperatively because the geometry has a low profile, and the epicondyles are covered by soft tissue [6, 7]. For correct femoral rotation in TKA, preoperative planning using computed tomography (CT) or magnetic resonance imaging (MRI) has been advocated by some surgeons [8, 9]. Although the PCA is the most apparent marker during surgery, the posterior condylar cartilage cannot be detected on CT [8]. In most osteoarthritic knees, the medial and lateral cartilages of the posterior condyle have different thicknesses because of asymmetric cartilage wear [10]. Previous studies using MRI have shown that posterior cartilage loss alters the apparent femoral condylar twist angle [11, 12]. These studies have proved that the angle of the TEA relative to the PCA is significantly greater in the presence of cartilage than in the absence of cartilage [11, 12]. However, in all these studies, the sample size was small, and the differences according to the mechanical axes and gender were not evaluated. Therefore, this study aimed to evaluate the effects of posterior condyle cartilage thickness on rotational alignment in the femoral component. The angle between the PCA and TEA was evaluated with respect to the presence of cartilage. In addition, the effect of the presence of cartilage on the femoral rotational axis was evaluated with respect to gender as well as varus and valgus conditions. We hypothesized that the effect of cartilage on femoral rotational alignment is influenced by these factors.

## Material and methods

We enrolled 684 patients with osteoarthritic knees in this study. Each patient was scheduled for primary TKA between January 2017 and December 2018 at our institute. Patients with a history of osteotomy of the affected knee or rheumatoid arthritis were not considered.

The subjects included 139 males and 531 females with a mean age of 69.8 ± 6.7 years. Their attributes are listed in Table 1. As a part of a standard preoperative protocol for patients with end-stage osteoarthritis who are scheduled to undergo TKA, MRI was conducted using a 1.5-T MRI scanner (Achieva 1.5 T; Philips Healthcare, Best, The Netherlands). A high-resolution 1-mm-thick slice in the sagittal plane of the tibiofemoral knee joint and a 5-mm-thick slice in the axial plane of the hip and ankle joints were obtained. For non-fat saturation imaging, MRI consisted of an axial proton density sequence, and a high-resolution setting was used for the spectral pre-saturation inversion recovery sequence (echo time, 25.0 ms; repetition time, 3590.8 ms; acquisition matrix, 512 × 512 pixels; number of excitations, 2.0; field of view, 140 × 140 mm). This method involved the use of patient-specific instruments and allowed us to effectively develop 3D reconstructed models.

**Table 1:** Comparison of the age and BMI between Korean males and females

Parameter	Whole patients (*n* = 670), mean ± SD (range)	Female (*n* = 531), mean ± SD (range)	Male (*n* = 139), mean ± SD (range)	*p* value
Age	69.8 ± 6.7 (32, 89)	69.9 ± 6.6 (32, 88)	69.5 ± 6.8 (52, 89)	0.56^n.s^
BMI	23.7 ± 3.6 (14.7, 41.3)	23.2 ± (16.6, 41.3)	23.9 ± 3.6 (14.7, 38.4)	0.08^n.s^

*n.s* non-significant

**Fig. 1:**
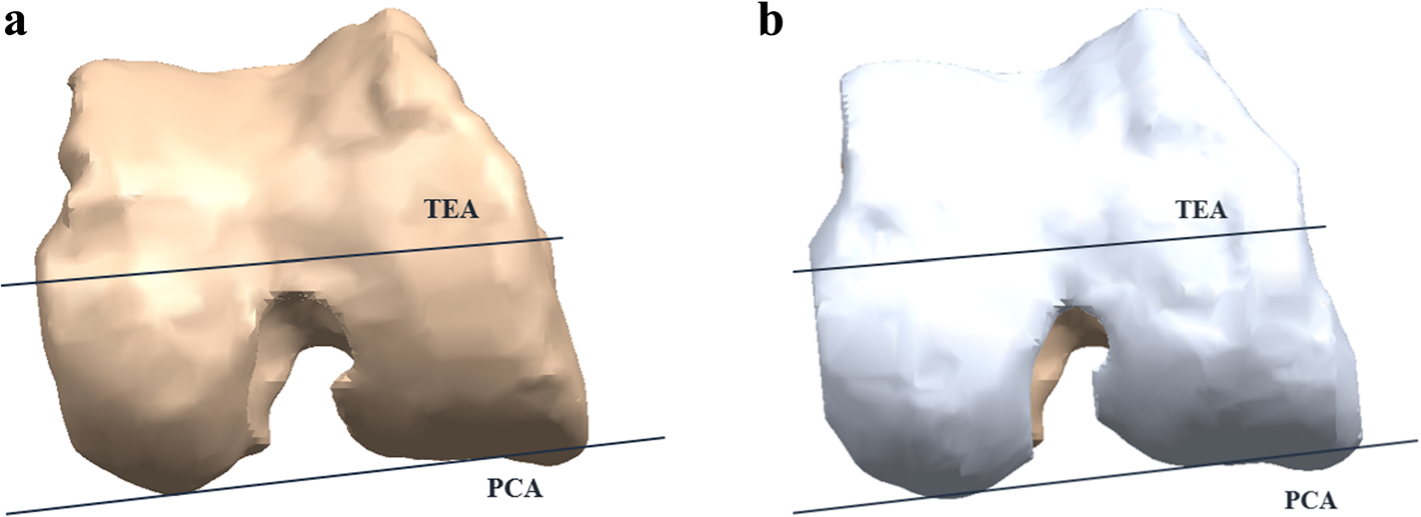
Schematic representation of the TEA and PCA axis of the femur **a** without and **b** with cartilage

The MRI data were used in two parts, and two 3D models were created for each patient. First, the bone was segmented and reconstructed as a 3D model (Fig. 1a). Then, the cartilage was segmented and combined with the bone model (Fig. 1b). From these data, we created a 3D model of a bone without cartilage. The second model was a 3D model of a bone with cartilage. These models were then imported into the design software. Then, anatomical markers were selected to create the anatomical axes: the femoral mechanical axis (FMA), the TEA, and the PCA. The TEA and PCA axes were projected onto the plane perpendicular to the FMA. The angle between the TEA and PCA axes was measured for comparison between the “with cartilage” and “without cartilage” models. The internal rotation direction of the PCA with respect to the TEA that induced external rotation of the femur was set as the positive (+) direction. Cartilage thickness was measured as the distance between the posterior condyle in the with cartilage and without cartilage models. The measurements were evaluated by a trained observer. To assess the intra- and inter-observer variability approximately (but not sooner than) 1 week after the initial measurements were taken, 50 female and 50 male participants again underwent 3D MRI conducted by the same observer and a second observer. The intra-observer agreement was 0.89, and the inter-observer agreement was 0.93, as calculated using the intra-class correlation method. The internal review board of our hospital (No.: 18-DR-02, Protocol No.: 3D-MRI according to sex_1.0) approved this study.

### Statistical analysis

All measurements are reported as means ± standard deviations. The independent *t* test was used to compare femoral rotational angles and cartilage thickness between females and males. The paired *t* test was used to compare femoral rotational angles and cartilage thickness according to the presence of cartilage. A *p* value of < 0.05 was considered statistically significant. SPSS for Windows version 12.0 (SPSS, Chicago, IL, USA) and G*Power (version 3.1.5) were used to perform the statistical analyses. The medial and lateral cartilage thicknesses in the entire patient with valgus knee were used as input parameters. The alpha value was 0.05, and the statistical power was 99.9%. The target power of previous studies was 80% [13, 14].

## Results

No significant differences were found between the gender in demographic characteristics, including age and BMI (Table 1). In all patients, the angle between the TEA and PCA was significantly greater in the presence of cartilage than in the absence of cartilage (*p* < 0.05) (Table 2). The angle between the TEA and PCA was 2.4° ± 0.9° in the presence of cartilage and 2.6° ± 1.0° in the absence of cartilage. This result was also found in the female group (*p* < 0.05). However, there was no significant difference in the angle between the TEA and PCA according to the presence of cartilage in the male group. The angle between the TEA and PCA was significantly greater in the presence of cartilage than in the absence of cartilage in the female varus group (*p* < 0.05). However, there was no significant difference in the angle between the TEA and PCA according to the presence of cartilage in the varus male group (Table 3). In addition, there was no significant difference in the angle between the TEA and PCA, regardless of the presence of cartilage, in the male and female valgus groups (Table 4). Lateral cartilage thickness was significantly greater than medial cartilage thickness in all patients, including females. There were no significant differences in medial and lateral cartilage thicknesses between genders (Table 5).

**Table 2:** Comparison of the TEA-PCA angle with and without cartilage between Korean males and females

Parameter	Whole patients (*n* = 670), mean ± SD (range)	Female (*n* = 531), mean ± SD (range)	Male (*n* = 139), mean ± SD (range)	*p* value
TEA-PCA (with cartilage)	2.4 ± 0.9 (− 2.6, 3.9)	2.4 ± 0.9 (− 1.6, 3.5)	2.5 ± 1.0 (− 2.6, 3.9)	0.35^n.s^
TEA-PCA (without cartilage)	2.6 ± 1.0 (− 1.6, 8.6)	2.6 ± 1.1 (− 0.6, 8.6)	2.5 ± 1.0 (− 1.6, 4.3)	0.46^n.s^
	< 0.05	< 0.05	0.93^n.s^	

*n.s* non-significant

**Table 3:** Comparison of the TEA-PCA angle with and without cartilage between Korean males and females in the varus knee

Parameter	Whole patients (*n* = 635), mean ± SD (range)	Female (*n* = 506), mean ± SD (range)	Male (*n* = 129), mean ± SD (range)	*p* value
TEA-PCA (with cartilage)	2.5 ± 0.9 (− 2.6, 3.9)	2.4 ± 0.9 (− 1.6, 3.5)	2.6 ± 0.9 (− 2.6, 3.9)	0.11^n.s^
TEA-PCA (without cartilage)	2.6 ± 1.0 (− 1.6, 8.6)	2.6 ± 1.1 (− 0.6, 8.6)	2.6 ± 1.0 (− 1.6, 4.3)	0.90^n.s^
	< 0.05	< 0.05	0.97^n.s^	

*n.s* non-significant

**Table 4:** Comparison of the TEA-PCA angle with and without cartilage between Korean males and females in the valgus knee

Parameter	Whole patients (*n* = 35), mean ± SD (range)	Female (*n* = 25), mean ± SD (range)	Male (*n* = 10), mean ± SD (range)	*p* value
TEA-PCA (with cartilage)	2.3 ± 1.0 (0.3, 3.4)	2.5 ± 0.9 (0.5, 3.3)	1.8 ± 1.3 (0.3, 3.4)	< 0.05
TEA-PCA (without cartilage)	2.2 ± 1.0 (0.4, 4.0)	2.4 ± 0.9 (− 0.6, 8.6)	1.6 ± 1.0 (0.5, 3.3)	< 0.05
	0.26^n.s^	0.40^n.s^	0.45^n.s^	

*n.s* non-significant

**Table 5:** Comparison of the medial and lateral cartilage thickness between Korean males and females

Parameter	Whole patients (*n* = 35), mean ± SD (range)	Female (*n* = 25), mean ± SD (range)	Male (*n* = 10), mean ± SD (range)	*p* value
Medial cartilage thickness	1.8 ± 0.4 (0.0, 5.0)	1.8 ± 0.4 (0.1, 5.0)	1.8 ± 0.5 (0.1, 5.0)	0.41^n.s^
Lateral cartilage thickness	2.2 ± 1.0 (0.4, 4.0)	2.4 ± 0.9 (− 0.6, 8.6)	1.6 ± 1.0 (0.5, 3.3)	0.34^n.s^
	< 0.05	< 0.05	0.06^n.s^	

*n.s* non-significant

## Discussion

We found that the angle of the TEA relative to the PCA was significantly greater in the presence of cartilage than in the absence of cartilage, especially in the female varus group. However, there were no such differences in the female valgus and male varus or valgus groups. Cartilage thickness in the lateral posterior femoral condyle was significantly greater than that in the medial posterior femoral condyle in all patients, including males.

Identification of the medial and lateral epicondyles during surgery is challenging [15, 16]. Furthermore, anatomical variability among patients, especially the degree of osteoarthritis, may impede correct measurement [17]. It is difficult to match the TEA observed on the radiograph with that seen intraoperatively [12]. For this reason, the PCA is used as a reference to find the TEA axis in the surgery using mechanical alignment. In the kinematic alignment, the posterior femoral cutting is conducted by the line parallel to PCA after correcting the cartilage and bone wear [18].

The relative angles between the TEA and PCA were 2.4° ± 0.9° and 2.6° ± 1.0°, in the presence and absence of cartilage, respectively. Asada et al. reported the mean relative angles of the TEA to the PCA to be 2.2° and 3.3° in the presence and absence of cartilage, respectively [19]. Both the angles and their trends were similar to our results. Tashiro et al. reported mean relative angles of 5.1° ± 2.1° and 6.8° ± 2.0° in the presence and absence of cartilage, respectively [11]. Thus, their study showed a trend similar to that seen in our study, but the higher values in their study indicated approximately 3° of additional external rotation [11]. The reason for this may be that anthropometric characteristics are related to many factors such as environmental, genetic, sociocultural, and lifestyle factors [20]. Owing to these variations among factors, the standard interpretation of values from different studies becomes difficult [20]. In addition, Tashiro et al. did not report the angle between the surgical and clinical TEA. However, the angle between the PCA and clinical TEA was 5.1° ± 2.1°. As the angle between the PCA and surgical TEA is approximately 3°, the PCA/TEA angle should be 2.1° ± 2.1° in the presence of cartilage.

In this study, it was found that the mean cartilage thickness is significantly different between the medial and lateral posterior femoral condyles in all patients. Cartilage thickness was significantly greater at the lateral posterior femoral condyle than at the medial posterior femoral condyle. The average difference in lateral and medial posterior thicknesses was 0.4 mm. This tendency was also found in a previous study [19]. The measurement of articular cartilage thickness in the posterior femoral condyle demonstrated that more cartilage wear occurred in the medial posterior condyle than in the lateral posterior condyle. These results also demonstrate that a more externally rotated femoral implantation is likely to occur on surgical planning using CT because the articular cartilage is disregarded [11]. Many surgeons determine anteroposterior cutting of the femur based on the posterior condyles and align the external rotation of the femoral component at a particular angle from the PCA to achieve a parallel line with the TEA [6, 21]. It has been reported that in addition to internal rotation, also excessive external rotation of the femoral component can worsen patellofemoral and tibiofemoral kinematics [22, 23]. In addition, external malrotation of the femoral component could increase polymeric wear, especially in early flexion. However, the restriction in rotation angles that a TKA can tolerate has not yet been defined [11].

Interestingly, the angle between the TEA and PCA was significantly greater in the presence of cartilage than in the absence of cartilage in the female varus group. In varus knees, the release of the medial ligament is often performed to balance medial and lateral tension in extension. However, a clinical study reported that the release of the posteromedial corner and superficial medial collateral ligament led to a 2.4° external rotation of the femur in knee flexion in cruciate-retaining total knee replacement [24]. If the neglection of cartilage thickness is added to the influence of such medial releases, then the femur can be as much as 4–5° externally rotated, leading to medial looseness in knee flexion. In most varus knees with arthritis, the articular cartilage of the medial posterior condyle is worn [12]. In contrast, the cartilage tends to be entirely preserved or only slightly degenerated in the lateral posterior condyle [12]. As this preserved cartilage cannot be detected on CT, the measured PCA tends to show internal rotation relative to that determined intraoperatively. For this reason, the condylar twist angle measured using CT would be overestimated when determining the appropriate rotational alignment of the femoral component. In some patients who have a greater amount of residual cartilage on the lateral side, using the PCA as the reference for placing the femoral component during surgery results in excess external rotation compared to that in cases in which the rotational angle is determined using radiographs, especially in female varus patients. This study provided evidence that residual cartilage may affect the setting of the rotational angle of the femoral component and defined the likely setting error encountered when using the PCA for alignment.

This study has some limitations. First, the population was restricted to Korean patients. The data used in this study may be typical for an Asian population, but anatomical differences may be noted in a Caucasian population. Second, in this study, MRI was used to develop 3D representations of the proximal tibia, which may have resulted in errors in the 3D models. Nevertheless, soft tissues such as the articular cartilage could be reconstructed using MRI, and the inaccuracy of the reconstruction could be reduced using a protocol described in a previous study [25]. Third, the MRI scans were re-assessed after insufficient time intervals; thus, memory effects could not be avoided. Finally, postoperative clinical outcomes were not considered in this study because patients who underwent TKA were not investigated.

## Conclusion

Our results indicate the importance of the effect of residual cartilage and the variability in individual condylar twist angles in determining the rotational angle. Surgical planning for TKA based on CT does not consider the articular cartilage, and this could lead to external rotational malposition of the femoral implant. Therefore, surgeons should consider the effect of the remaining posterior condylar cartilage to prevent over-rotation of the femoral component, especially in female varus knees.

## Data Availability

The data are available from the corresponding author upon reasonable request.
